# The Modern Dentist from a Medical Stand-Point

**Published:** 1902-09

**Authors:** William Knight

**Affiliations:** Cincinnati, Ohio


### THE MODERN DENTIST FROM A MEDICAL STAND-POINT.[1]

BY DR. WILLIAM KNIGHT, CINCINNATI, OHIO.

ONE result of the Baconian method of research has been to enlighten both the scientific and the professional mind. Previous to the time of Bacon, scholastic arrogance prevailed, and the votaries

[1] Read before the Section on Stomatology of the American Medical Association, at Saratoga Springs, June, 1902.

of the then dawning sciences kept themselves aloof from a large portion of their fellow-beings, whom they regarded with the greatest contempt or indifference. Scholasticism, long decaying, received its death-blow from Bacon. It, however, struggled desperately against its destruction, and so persistent has been the contest, that at the present day there remains a remnant of that haughty arrogance. But that is slowly disappearing before the radiance that is glowing from the secrets of ethical and physical laws that are now being revealed to man. This advance in knowledge has gradually developed a fraternal feeling among the various investigators in not only the closer but also the more distantly allied branches of science. This accession in science has necessarily enlarged the views of man, and consequently has made inroads into that seclusiveness that had grown and encircled most of the professions, and particularly the medical profession.

It would be interesting, indeed, to trace historically the gradual crumbling of those seclusive walls, built as they were of arrogance and prejudice. But that is not our present purpose. We desire simply to emphasize the fact that it is due to this general advancement in science that we have to-day a medical profession that is liberal and democratic, and that is extending the hand of fellowship to every one engaged in the healing art, irrespective of the specialism that he may have chosen. As thus, when, in 1882, a few of the prominent practitioners of dentistry desired that a section representing their branch of surgery be created by the American Medical Association, their request was granted, and a section on stomatology was added to the existing sections of that representative body. This professional recognition of dental surgeons entitled them to the privileges, advantages, and courtesies due to any other member of the American Medical Association. This affiliation should be an incentive to members of local and State dental societies to occasionally discuss subjects that have a general bearing upon medical questions. This is the method followed by other specialisms, as, for instance, those of ophthalmology and dermatology.

I believe that a dental student, when matriculating at a dental college, should be impressed that he is about to enroll himself as an aspirant to membership in what is termed a learned profession. He would thus, at the beginning of his studies, realize that his curriculum must necessarily be a comprehensive one, embracing all of the studies pertaining to the profession of which he hopes to become a member. I believe few of those who have given anatomical lectures to dental students will dissent from the statement that, with few exceptions, it is difficult to impress the dental student with the importance of acquiring a practical knowledge of anatomy. This would not be the case if they would view the matter as they should, —from a medical stand-point. Last year, when in London, I was informed by one of the examiners for English qualifications for American dental graduates that most of the candidates were quite defective in anatomical and physiological attainments. The reason for this lies in the fact that heretofore the dental student has not realized how necessary it is for him to acquire a practical knowledge of these two studies. The progressive dental surgeon of to-day is conscious that his knowledge of anatomy and pathological anatomy is essential to him, for he sees, almost daily, diseased conditions in the mouth and its surroundings, the character of which can only be understood by applying the teachings of these two sciences. The dentist not infrequently observes morbid conditions in the mouth that are produced by some constitutional disturbance. He thus finds himself confronting questions pertaining to general medicine. For instance, he may find healthy teeth loosened in their sockets, perhaps an entire row of them. No other morbid conditions are apparent; there are no symptoms, nor have there been, of Rigg's disease; there is no absorption of the alveolar processes, no shrinking of the gum tissues. An examination is made of the urine, and sugar is detected; the diagnosis of diabetes is made, and the indications for treatment become clear. In this connection the following cases present features of interest:

An English army officer suffered from a severe and continuous pain in his left eye. The oculist whom he consulted, after exhausting all means of relief known to him, and affording none, decided upon enucleation. The operation was performed, but, unfortunately, the right, the unaffected eye, was removed by mistake. There was no relief obtained for the left eye by the operation. It was now decided that the mouth should be carefully examined, which would have been done before the right eye was removed if a dental surgeon had been consulted. The examination of the mouth resulted in the discovery of a carious tooth, and, although the tooth gave but slight discomfort, it was extracted. The result was most fortunate. The pain in the eye abated at once, and in the course of a few days disappeared entirely.

A few years ago I treated a young man for clonic spasms of the upper right eyelid. After some ten days' treatment, the condition remaining the same, I advised him to consult his dentist. He smiled at this, but did as I advised him. He returned a few days afterwards entirely relieved of the annoying affection. His dentist had discovered a small painless cavity in an upper molar tooth, which he treated. The clonic spasms of the eyelid in this case were evidently associated with the carious tooth, and afford a good illustration of some reflex act excited through the sensitive filaments of the second division of the fifth cranial nerve, affecting thereby some filaments of the seventh cranial, *a motor nerve.*

These cases are illustrated, and indicate that consultation should be more frequent between the dental surgeon and the general practitioner, especially with the ophthalmic surgeon. A large number of cases could be cited to prove the dependency of one part of the human body upon another part, and if the teeth were selected for this purpose, a most interesting group exemplifying this could be collected.

The teeth, although anatomically regarded as dermal appendages, have a very close functional relationship with several of the cranial, and more remotely with some of the cervical spinal nerves. How frequently are inflammatory conditions seen in the face, neck, and even the external chest, that owe their origin to disturbed nutrition, brought about by irritation starting in a diseased tooth. In this connection a case recorded by John Hilton in his classical work, "Rest and Pain," is worthy of full quotation.

"A professional friend had an enlarged gland below the external ear, the real cause of which was not quite apparent, and so he requested me to look at it. There was a slight discharge of morbid secretion in the auditory canal. We argued together, and I said, 'Very likely it may be the result of a decayed tooth; irritation from it may be conveyed to the auditory canal and induce morbid secretion. That morbid secretion may produce slight excoriation, and that excoriation, aided by lymphatic absorption, may explain the existence of the enlarged gland.' The tooth was extracted, all the other local morbid conditions disappeared, and there was no recurrence of the local symptoms."

This case proves that irritation of a nerve, the fifth cranial in this instance, is sufficient to lead to more or less change in function and structure, and that morbid influence may, after a time, induce a deterioration resulting in ulceration, etc. How important, also, it is that the dental surgeon should be able to diagnose in an early stage the various neoplasms so frequently seen growing in the mouth, lips, jaws, and tongue! Especially is this of great moment in instances of malignancy arising in the regions mentioned. It not infrequently happens that a patient having an incipient malignant growth, of which he is not conscious, has pain in a tooth, the pain in the tooth being caused by the presence of the small malignant tumor. The failure of the dentist, whom the sufferer consults, to recognize the existing conditon may result in the destruction of the patient. Two instances of this kind have come under my observation during the past few years. In both cases the malignant growth was sarcomatous. One originated in the antrum, the other made its first appearance in the molar region of the inferior maxiliary bone. In each case the tooth had been extracted for the relief of pain. Some months afterwards, when the patients came under my observation, infiltration of the neighboring soft parts had occurred to such an extent that a successful result could not be expected to follow even a most radical operation. On the urgent solicitation, however, of the patient suffering with a sarcoma of the upper jaw, I agreed to operate, with the understanding that in all probability the affection would return. At the time of the operation I removed half of the upper jaw, together with a large quantity of sarcomatous tissue from the surrounding parts. Growth returned after several months, the patient dying a few months afterwards from exhaustion. I refused to operate in the other case, as the floor of the mouth, the gum, and the cheek were extensively involved. I, however, used injections of Colly's serum (the streptococcus erysipelatus prodigiosum), so highly recommended in inoperable cases of sarcoma, with the result, however, of adding to the sufferings of the patient, who succumbed to his affection six months after I first saw him.

Much more frequent than neoplasms and inflammatory affections that have their origin from dental irritation are those instances that are purely reflex in their character, and that are excited through irritation of the dental branches of the fifth cranial nerve. Most of these cases can only be diagnosed by applying the general principles of medicine to their elucidation; the constitutional peculiarities or taints of the individual must be considered. A rheumatic, gouty, or specific habit must be recognized before rational treatment in any given case can be decided upon.

For the reasons given in these brief remarks, it must be apparent that the dental surgeon of to-day must have a knowledge of the principles of medicine, and by virtue of his professional attainments he becomes a member of that great brotherhood, the medical profession, whose mission it is to relieve human sufferings.

